# Thyroïdite de Riedel

**DOI:** 10.11604/pamj.2015.20.290.6593

**Published:** 2015-03-25

**Authors:** Madiha Mahfoudhi, Khaled Khamassi

**Affiliations:** 1 Service de Médecine Interne A, Hôpital Charles Nicolle, Tunis, Tunisie; 2 Service ORL, Hôpital Charles Nicolle, Tunis, Tunisie

**Keywords:** thyroïdite de Riedel, dysphagie, examen anatomopathologique, Riedel thyroiditis, dysphagia, pathological examination

## Image en medicine

La thyroïdite de Riedel représente la forme la plus rare de thyroïdite chronique. Elle est caractérisée par l'apparition d'une fibrose infiltrative et extensive entraînant une destruction de la thyroïde et s’étendant aux organes de voisinage. Son étiopathogénie demeure inconnue. Son diagnostic est anatomopathologique. Sa prise en charge reste discutée. Patiente âgée de 47 ans, diabétique a consulté pour une tuméfaction basi-cervicale antérieure évoluant depuis 2 mois, rapidement évolutive, associée à une dysphagie et une dyspnée d'effort, sans dysphonie ni signes de dysthyroïdie. L'examen physique a révélé une formation basi-cervicale dure, mal limitée, indolore, mobile à la déglutition, de 10 cm de grand axe. Les aires ganglionnaires étaient libres. Le bilan thyroïdien était normal. L’échographie cervicale a retrouvé une thyroïde augmentée de taille, hétérogène sans nodules décelables. La TDM cervicale a objectivé une hypertrophie hypodense de la thyroïde, sans extension intra-thoracique. Cette hypertrophie englobait les axes carotidiens primitifs droits et gauches. Plusieurs diagnostics ont été évoqués particulièrement le cancer thyroïdien invasif (carcinome anaplasique), la forme fibreuse de la thyroïdite d'Hashimoto et le lymphome thyroïdien. En per-opératoire, la glande thyroïde était dure, fibrosée, inextirpable et englobait la trachée et les axes vasculaires du cou des 2 côtés. Une biopsie en quartier d'orange a été alors réalisée. L'examen anatomopathologique a conclut à une thyroïdite de Riedel. La patiente a été adressée en endocrinologie où elle a été mise sous corticoïdes L’évolution était favorable avec diminution de la taille du goitre et disparition des signes de compression.

**Figure 1:**
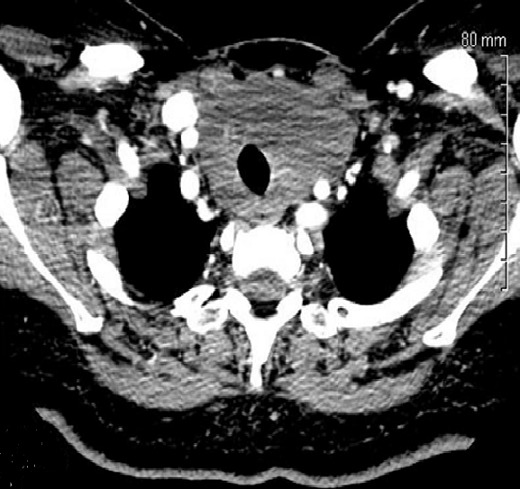
TDM (coupe axiale): hypertrophie hypodense de la glande thyroïde

